# Increasing Receipt of High-Tech/High-Cost Imaging and Its Determinants in the Last Month of Taiwanese Patients With Metastatic Cancer, 2001–2010: A Retrospective Cohort Study

**DOI:** 10.1097/MD.0000000000001354

**Published:** 2015-08-14

**Authors:** Tsang-Wu Liu, Yen-Ni Hung, Thomas C. Soong, Siew Tzuh Tang

**Affiliations:** From the National Institute of Cancer Research, National Health Research Institutes, Miaoli County (T-WL); School of Gerontology Health Management and Master's Program in Long-Term Care, College of Nursing, Taipei Medical University (Y-NH); Department of Radiology, Koo Foundation Sun Yat-Sen Cancer Center, Taipei (TCS); and Chang Gung University, School of Nursing, Kwei-Shan, Tao-Yuan, Taiwan, R.O.C. (STT).

## Abstract

One strategy for controlling the skyrocketing costs of cancer care may be to target high-tech/high-cost imaging at the end of life (EOL). This population-based study investigated receipt of high-tech/high-cost imaging and its determinants for Taiwanese patients with metastatic cancer in their last month of life.

Individual patient-level data were linked with encrypted identification numbers from computerized administrative data in Taiwan, that is, the National Register of Deaths Database, Cancer Registration System database, and National Health Insurance claims datasets, Database of Medical Care Institutions Status, and national census statistics (population/household income). We identified receipt of computerized tomography (CT), magnetic resonance imaging (MRI), positron emission tomography (PET), and radionuclide bone scans (BSs) for 236,911 Taiwanese cancer decedents with metastatic disease, 2001 to 2010. Associations of patient, physician, hospital, and regional factors with receiving CT, MRI, and bone scan in the last month of life were evaluated by multilevel generalized linear-mixed models.

Over one-third (average [range]: 36.11% [33.07%–37.31%]) of patients with metastatic cancer received at least 1 high-tech/high-cost imaging modality in their last month (usage rates for CT, MRI, PET, and BS were 31.05%, 5.81%, 0.25%, and 8.15%, respectively). In 2001 to 2010, trends of receipt increased for CT (27.96–32.22%), MRI (4.34–6.70%), and PET (0.00–0.62%), but decreased for BS (9.47–6.57%). Facilitative determinants with consistent trends for at least 2 high-tech/high-cost imaging modalities were male gender, younger age, married, rural residence, lung cancer diagnosis, dying within 1 to 2 years of diagnosis, not under medical oncology care, and receiving care at a teaching hospital with a larger volume of terminally ill cancer patients and greater EOL care intensity. Undergoing high-tech/high-cost imaging at EOL generally was not associated with regional characteristics, healthcare resources, and EOL care intensity.

To more effectively use high-tech/high-cost imaging at EOL, clinical and financial interventions should target nonmedical oncologists/hematologists affiliated with teaching hospitals that tend to aggressively treat high volumes of terminally ill cancer patients, thereby avoiding unnecessary EOL care spending and transforming healthcare systems into affordable high-quality cancer care delivery systems.

## INTRODUCTION

Cancer care costs are escalating, threatening national economies, and the long-term sustainability of healthcare systems internationally.^1,2^ Controlling these skyrocketing costs depends on identifying unnecessary care without meaningful benefits.^2^ One potential target is high-tech/high-cost imaging.^3^ Indeed, diagnostic imaging use and expensive tests have dramatically increased over the past decade, contributing to soaring healthcare costs and outstripping other sectors of healthcare expenditures.^4^

Correspondingly, increased high-tech/high-cost imaging and subsequent treatments to manage health problems have not clearly benefited cancer patients at end of life (EOL),^3,5^ contributing instead to their highest cancer-care costs.^6^ Indeed, one-third of EOL care expenditures are concentrated in the last month^7^ when decedents’ cancer care becomes increasingly aggressive.^8^ Therefore, healthcare systems must ensure the appropriate and sustainable growth of advanced imaging in cancer care overall, and in EOL cancer care specifically.^3^

Nonetheless, relatively few studies have investigated high-tech/high-cost imaging use beyond staging and not for screening/posttreatment surveillance in cancer patients generally^4,5,9,10^ and at EOL specifically.^11^ These studies^4,5,9–11^ focused on Medicare beneficiaries with selected cancers, for example, lung, breast, colorectal, and prostate cancer. Furthermore, they primarily investigated trends in high-tech/high-cost imaging use and seldom explored patient, physician, hospital, and regional factors^12^ contributing to receiving high-tech/high-cost imaging. Therefore, this population-based study investigated receipt of high-tech/high-cost imaging and its determinants for patients with distant metastatic disease on all ages and disease groups of cancer in their last month of life. We hypothesized that receiving high-tech/high-cost imaging in the last month of life would be associated with patient demographics and disease characteristics, physician specialty, and characteristics, healthcare resources, and EOL care practice patterns at both the hospital and regional level.

## METHODS

### Design and Sample

For this retrospective cohort study, individual patient-level data were linked with encrypted identification numbers from computerized administrative data, that is, the National Register of Deaths Database (NRDD), Cancer Registration System (CRS) database, and National Health Insurance (NHI) claims datasets, Database of Medical Care Institutions Status, and national census statistics (population/household income). Taiwan's government monitors completeness and accuracy of these databases. All deaths are required to be registered in Taiwan and all death certificates issued by physicians are centralized to the Department of Health. Cause-of-death information of the NRDD is highly accurate for malignant neoplasms (kappa = 0.94 with medical record reviews).^13^

The Taiwan Cancer Registry, a population-based cancer registry, was founded in 1979. Hospitals with >50 beds which provide outpatient and inpatient cancer care are required to report all newly diagnosed malignant neoplasms to the registry. The CRS included 97.3% of incident cancer cases, with 97.0% completeness and 91.1% accuracy.^14^

Taiwan's government-run NHI offers universal coverage and free access to fee-for-service comprehensive health services. Copayment is waived for patients with identified major diseases, including malignancy. By 2010, the NHI had enrolled 99.6% of the 23 million residents in Taiwan.^15^ The quality of NHI claims dataset is ensured by extensive and systematic processes, including clinical specialists routinely crosschecking chart reviews to ensure accuracy in diagnostic coding, comorbidities, and healthcare resources utilization. NHI datasets have been validated,^16,17^ and information on diagnoses, healthcare resources utilization, and EOL care used in epidemiologic and clinical research, and information was of high quality.^16–19^ The Database of Medical Care Institutions Status provided information on hospital characteristics and healthcare resources for each hospital and region.

**FIGURE 1:**
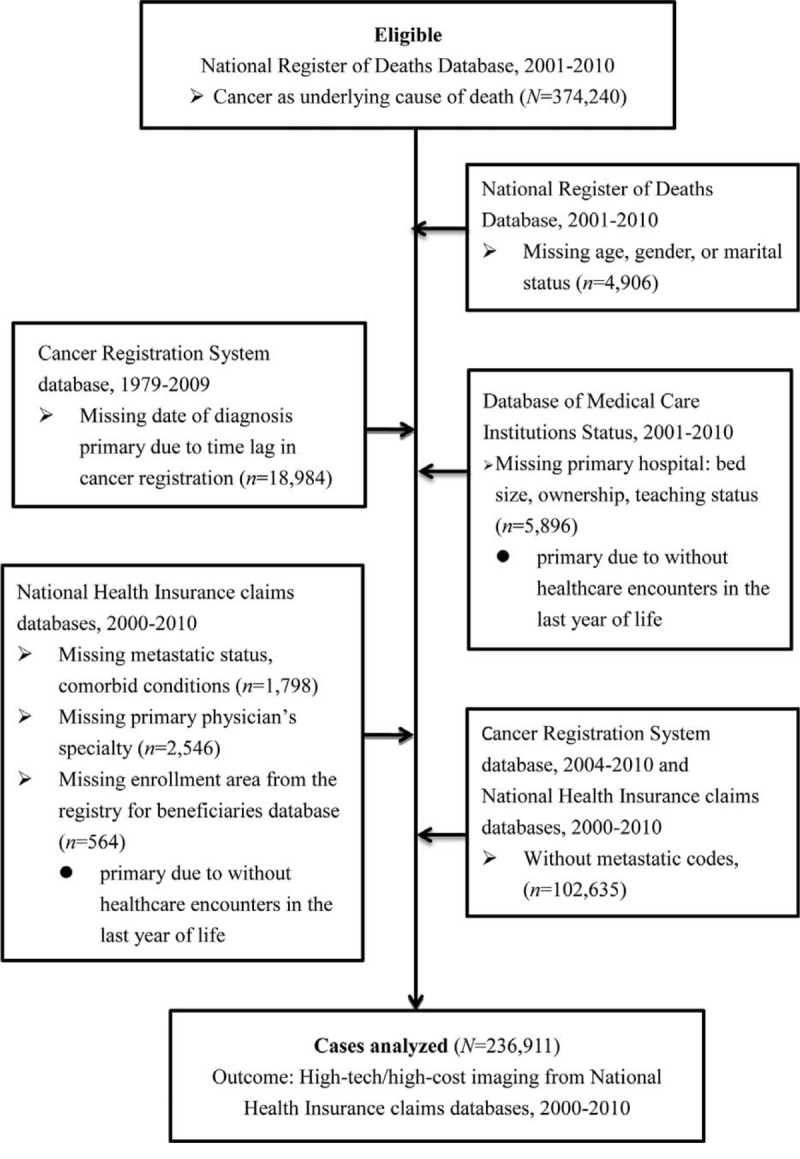
Patient identification algorithm.

The NRDD identified 374,240 cancer deaths from 2001 to 2010; 34,694 were excluded from our analyses primarily due to missing information on date of cancer diagnosis by the time lag of the CRS and characteristics of patients’ primary hospital because some patients did not have healthcare encounters in their last year of life. To focus on patients at EOL, we identified 236,911 cancer decedents with metastatic disease. Metastatic status was identified by at least 1 inpatient or 2 outpatient claims with ICD-9 codes 196.xx–199.xx at least 30 days apart^20^ during patients’ last year of life or by stage IV indicated in the CRS datasets since 2004. The detailed patient identification algorithm is shown in Figure 1. For patients’ demographics, disease characteristics, primary hospital and regional healthcare resources, and EOL care practice patterns, see Table 1. This study was approved by the Chang Gung Memorial Hospital Institutional Review Board, which waived the requirement of consent. This study followed the stands for The Strengthening the Reporting of Observational Studies in Epidemiology guidelines.

**TABLE 1:**
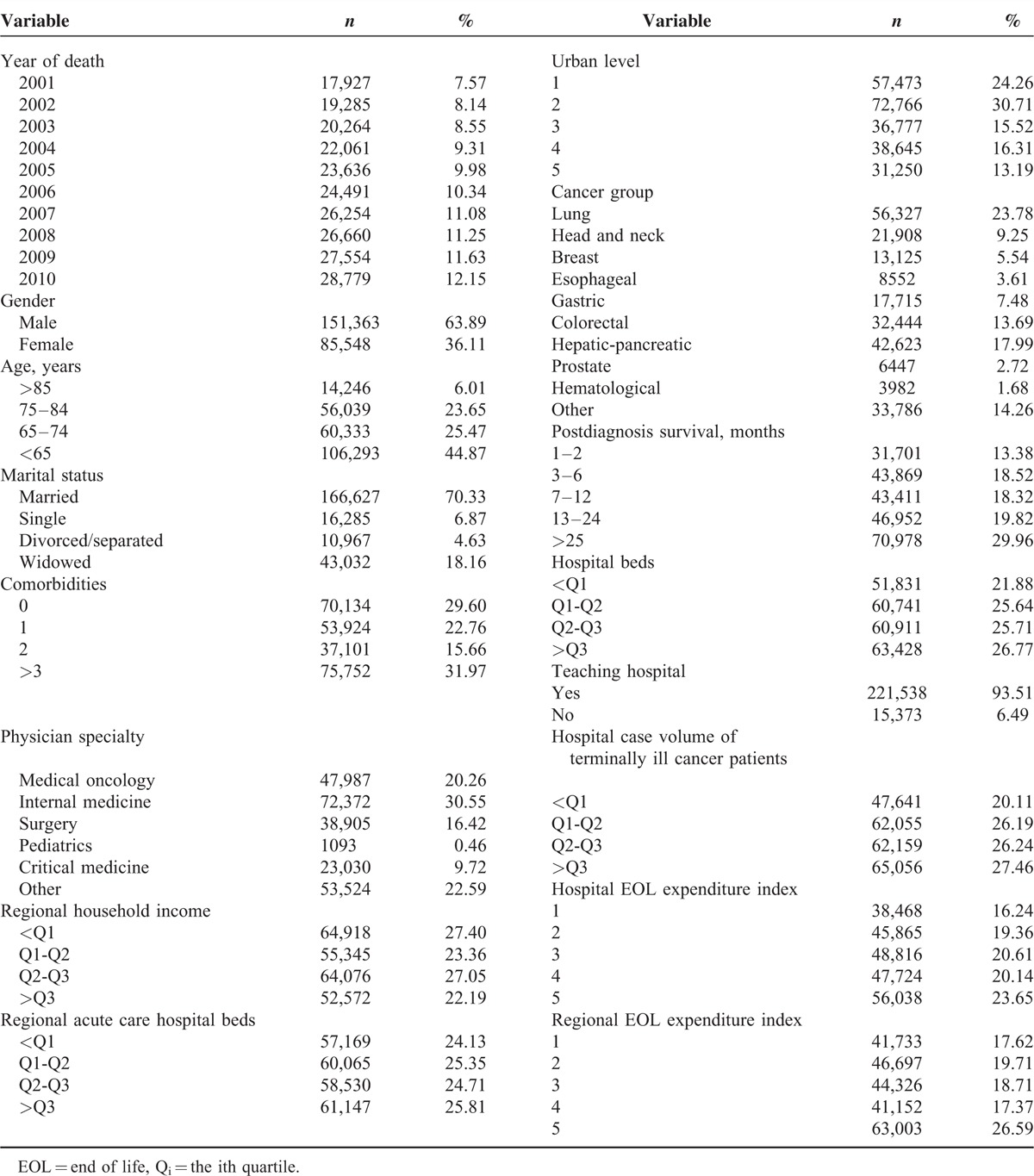
Characteristics of Participants and Their Primary Hospital and Region

### Measures

#### Outcome Variable

Receiving at least 1 high-tech/high-cost imaging procedure was identified by billing codes on NHI claims datasets specifically for computerized tomography (CT), magnetic resonance imaging (MRI), positron emission tomography (PET), and radionuclide bone scans (BSs) in patients’ last month of life. CT, MRI, and BS have been reimbursed by the NHI since its inception in 1995, whereas PET has been available since 2004.

#### Independent Variables

Independent variables, based on a conceptual framework for determining treatment intensity for seriously ill patients,^21^ were patient socio-demographics and disease characteristics; primary physician's specialty; and characteristics, healthcare resources, and EOL care practice patterns for the primary hospital and its region.

#### Patient socio-demographics

Patient socio-demographics included gender, age, marital status, and residential urbanization level. Urbanization level was classified by population density and proportions of the population with at least a college education, over age 65, or agricultural workers^22^ to reflect the social-economic status of each city/county. Residential urbanization level was stratified from 1 (most urbanized) to 5 (least urbanized)^22^ by combining the original levels 5 to 8 (remote and rural areas) to avoid too few patients at these levels.^22^

#### Patient disease characteristics

Patient disease characteristics included comorbidities, cancer diagnosis, and postdiagnosis survival time. Comorbidities were identified from ICD-9 codes for primary and secondary diagnoses, excluding cancer-related codes in NHI claims for inpatients and outpatients during their last year. These codes were used to calculate the Deyo–Charlson comorbidity index,^23^ categorized as 0, 1, 2, or ≥3 comorbid conditions. Diagnosis and date of diagnosis were identified from the CRS. Postdiagnosis survival, the interval between dates of diagnosis and death, was categorized into 1 to 2, 3 to 6, 7 to 12, 13 to 24, and >25 months.

#### Primary physician's specialty

Primary physician's specialty was retrieved from NHI claims by physician specialty code and categorized into medical oncology (including hematology) and 4 other specialties commonly caring for cancer patients.

#### Primary hospital characteristics, healthcare resources, and EOL care practice patterns

We attributed each patient's medical care to the hospital providing the most hospitalizations in the patient's last year of life. Characteristics and healthcare resources included teaching status and acute care bed size, which was grouped into the hospital's quartile ranking for bed size. EOL care practice patterns were characterized by the case volume of terminally ill cancer patients and EOL care intensity. Case volume of terminal cancer patients was computed as the annual number of cancer patients admitted during their last 6 months of life^24^ and categorized into quartiles. EOL care intensity, considered as an indicator of how aggressively a hospital treats similarly ill patients,^25^ was defined as total spending on medical care (including both cancer- and noncancer-related) for patients at EOL and assessed using a Medicare-spending measure, the end-of-life expenditure index (EOL-EI).^25^ Hospital EOL-EI was calculated as age–sex-adjusted mean spending on inpatient, emergency department, and outpatient services for cancer patients in their last 6 months of life. EOL care expenditures were computed for individuals and aggregated to the primary hospital. We grouped hospitals into quintiles of increasing EOL care intensity.

#### Primary hospital's regional characteristics, healthcare resources, and EOL care practice patterns

Patients were assigned to a region including the county/city of their primary hospital's location. Regional annual household incomes were imputed from national statistics by assigning cancer decedents’ primary hospital the household median income for the hospital's region. Regional household incomes were stratified by quartile. Regional healthcare resources were measured by the number of acute care beds, categorized into quartiles of beds per 10,000 population. Regional EOL care practice patterns (EOL care intensity) were measured by regional EOL-EI,^25^ calculated for each hospital as described for the primary hospital level, and aggregated to the region for each cancer decedent's primary hospital. The mean regional-adjusted spending for cancer decedents in their last 6 months of life was further stratified by quintile.

#### Statistical Analysis

Associations of patient, physician, hospital, and regional factors with receiving CT, MRI, and bone scan in the last month of life were evaluated by multilevel generalized linear-mixed models^26^ using a logit link function by SAS GLIMMIX procedure. Each patient's primary hospital was used as a random effect with patient, physician, hospital, and regional factors as fixed effects to account for correlation in the error term due to individuals clustering in the same hospital and nesting of hospitals in their regions. Healthcare resources and EOL care practice patterns for the primary hospital and its region were checked for multicollinearity; variance inflation factors ranged from 1.11 to 7.04, indicating no severe multicollinearity.^27^ The regression parameter for each independent variable was exponentiated into adjusted odds ratio with 95% confidence interval. Given our large population-based sample and multiple tests performed in multilevel generalized linear-mixed modeling, significance is reported at *P* < 0.001.

## RESULTS

**FIGURE 2:**
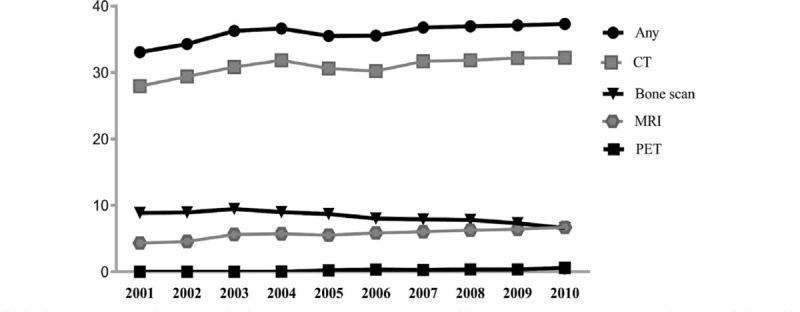
Trends in receipt of high-tech/high-cost imaging in the last month of Taiwanese patients with metastatic cancer, 2001 to 2010.

Subjects’ rates of receiving CT (27.96%–32.22%), MRI (4.34%–6.70%), and PET (0.00%–0.62%) in their last month of life increased over the 10-year study period, whereas receiving BS decreased (9.47%–6.57%) after 2009 (Figure 2) (all *P* < 0.001). Over one-third (average [range]: 36.11% [33.07%–37.31%]) of Taiwanese cancer patients who died with metastatic disease received at least 1 of the 4 high-tech/high-cost imaging modalities in their last month of life. Since PET was not introduced into Taiwan's NHI until 2004 and relatively few subjects received PET at EOL, determinants of its use were not identified.

Receiving CT and BS was significantly more likely for male patients (Table 2). Receiving CT, MRI, and BS increased consistently with decreasing age. MRI and BS receipt were less likely for unmarried cancer patients, whereas undergoing CT and BS was more likely for patients residing in less urbanized areas, reaching significance for the 2 least urbanized areas.

**TABLE 2:**
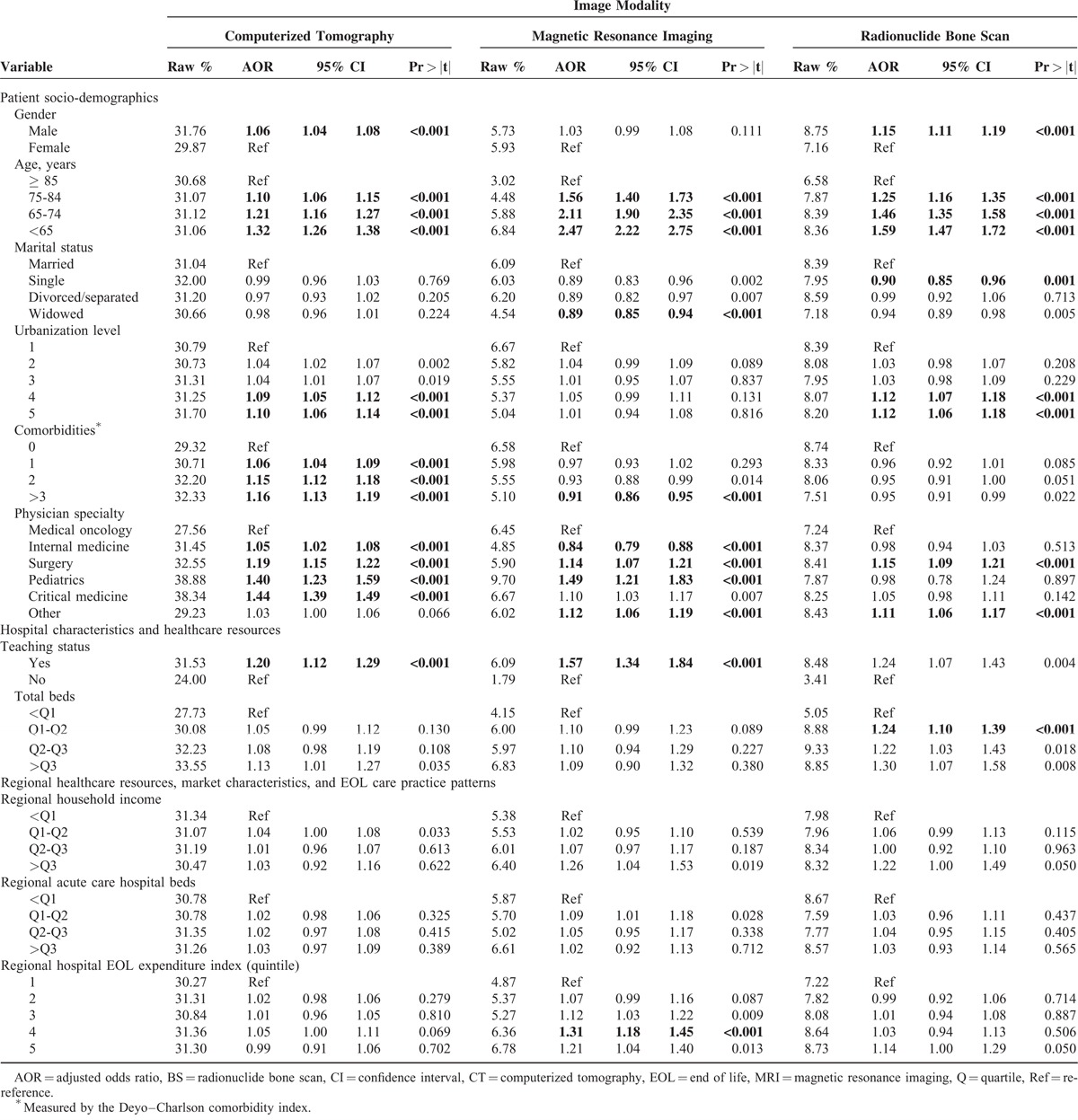
Determinants of CT, MRI, and BS Use in the Last Month for Taiwanese Patients With Metastatic Cancer, 2001 to 2010

**FIGURE 3:**
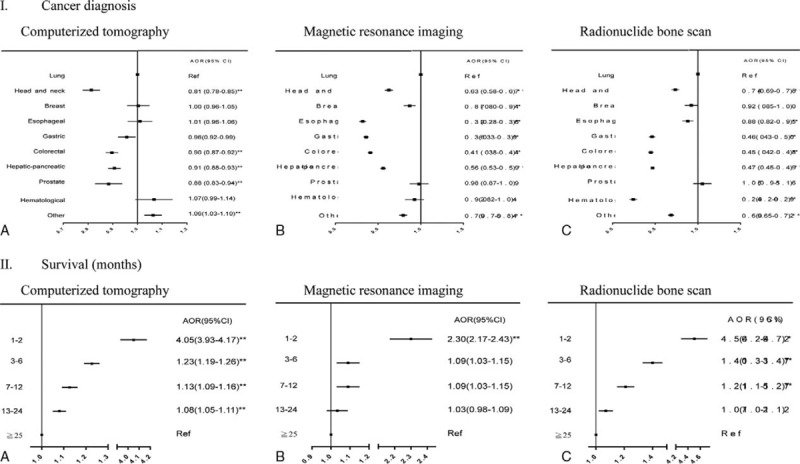
Associations of disease characteristics with receiving high-tech/high-cost imaging in cancer patients’ last month of life.

Receiving high-cost/high-tech imaging at EOL was significantly associated with patient disease characteristics. Lung cancer patients were significantly more likely than patients with cancer of the head and neck, esophagus, stomach, colon-rectum, and liver or pancreas to receive MRI and BS in their last month of life (Figure 3I), whereas patients with cancer of the head and neck, colon-rectum, liver or pancreas, and prostate were significantly less likely than lung cancer patients to undergo CT in their last month of life. Patients with hematological malignancies were significantly less likely than lung cancer patients to receive BS in their last month of life only. Patients who died within 1 and 2 years postdiagnosis were more likely to receive MRI/BS and CT, respectively, than those who lived longer after diagnosis (Figure 3II). More comorbidities were associated with higher likelihood of receiving CT at EOL, whereas the opposite pattern was observed for MRI (Table 2).

**FIGURE 4:**
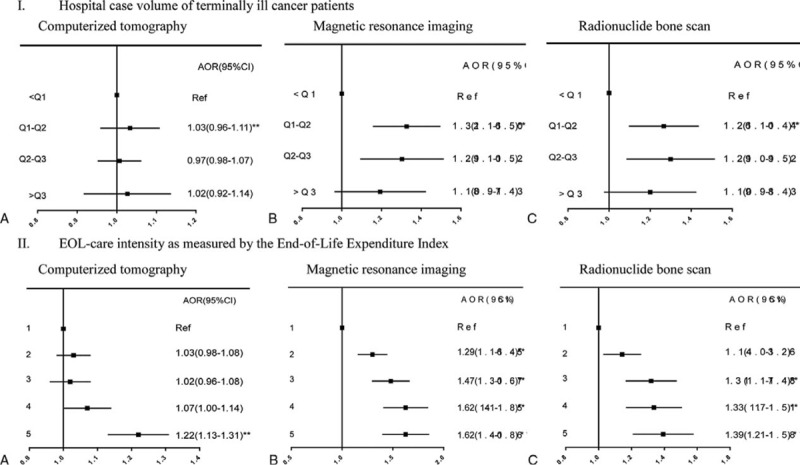
Associations of primary hospital's end-of-life care practice patterns with receiving high-tech/high-cost imaging in cancer patients’ last month of life.

Receiving high-tech/high-cost imaging in the last month of life was associated with patients’ physician specialty and primary hospital characteristics, healthcare resources, and EOL care practice patterns. Oncologists/hematologists were generally less likely than other physician specialists to provide CT, MRI, and BS in subjects’ last month of life (Table 2). Increased likelihood of undergoing CT and MRI at EOL was associated with receiving care at teaching hospitals, whereas increased receipt of BS was associated only with more acute care hospital beds. Receiving care at hospitals with larger case volumes of terminal cancer patients (Figure 4I) and higher EOL care intensity (indicated by the EOL-EI) (Figure 4II) predisposed patients to receive MRI and BS in their last month of life, whereas undergoing CT was only significantly, positively associated with receiving care at hospitals with the highest EOL-EI.

However, undergoing high-tech/high-cost imaging at EOL was not associated with regional characteristics, healthcare resources, and EOL care practice patterns, except for regional EOL care intensity (Table 2). Receiving care at hospitals in a region with more intense EOL care was generally associated with a higher propensity to receive high-tech/high-cost imaging at EOL, reaching significance at the 4th EOL expenditure quintile for receiving MRI by our strict criterion.

## DISCUSSION

Over one-third (average [range]: 36.11% [33.07%–37.31%]) of Taiwanese patients with metastatic cancer received at least 1 high-tech/high-cost imaging modality in their last month of life (31.05% [27.96%–32.22%], 5.81% [4.34%–6.70%], 0.25% [0.00%–0.62%], and 8.15% [6.57%–9.47%], for CT, MRI, PET, and BS, respectively). Corresponding figures for Medicare beneficiaries with stage IV breast, colorectal, lung, or prostate cancer were 34.3% (30.6%–36.8%), 29.3% (25.7%–31.1%), 9.1% (3.9%–11.1%), 1.1% (0.2%–1.3%), and 6.0% (3.6%–8.3%), respectively.^11^ This US-based study^11^ examined only patients >65-years old, whereas our study population included all ages. Since older patients receive less aggressive EOL care^8^ and are less likely to undergo high-tech/high-cost imaging,^5,28^ Taiwanese stage IV cancer patients’ high-tech/high-cost imaging receipt may be at least comparable to or less than that of American cancer patients in their last month of life, despite all Taiwanese cancer patients having free access to imaging procedures with copayments waived.

Similar to Medicare cancer patients, Taiwanese cancer patients underwent increasing trends of CT,^4,9,11^ MRI,^4,9,11^ and PET,^4,9–11,28^ but decreasing BS.^9,11^ The declining trend in BS for our subjects, along with the increasing trends in CT, MRI, and PET use, suggests that clinicians are using more expensive alternatives (CT, MRI, and PET) to detect osseous metastatic disease even in patients’ last month of life.^9^ Using high-tech imaging to detect metastatic disease may have important management and prognostic implications early in the cancer trajectory. However, in patients’ last month of life, when death is imminent despite its exact date being unknown, using expensive high-tech imaging techniques to detect new metastatic foci hardly leads to timely and beneficial disease management. Indeed, the current clinical practice of lengthy multifractionated radiotherapy for bone metastasis^29–31^ results in treatment being prematurely terminated without symptom improvement^30,32^ due to patient decline or death, and often increases healthcare costs.^3,11^

As reported, we found that high-tech/high-cost imaging use at EOL for CT, MRI, and BS was consistently facilitated by younger age,^5,28^ being married,^28^ and lung cancer diagnosis.^4,9,11^ Our results also confirm the frequently documented relationships of greater likelihood of imaging with male gender^33^ and rural residence.^34,35^ However, our findings on these relationships were only significant for CT and BS use.

Receiving high-tech/high-cost imaging in Taiwanese cancer patients’ last month of life was significantly more likely for those who died within 1 to 2 years of diagnosis than those who survived longer (Figure 3II), consistent with more frequent imaging for Medicare beneficiaries who survived <1-year postdiagnosis.^11^ This finding may reflect the attitude of “coming to terms with one's disease,” which is more likely in patients who have fought cancer for a longer time. Furthermore, high-tech/high-cost imaging may frequently be used to diagnosis advanced cancer but patients may then die within 1 to 2 months due to aggressive disease. However, our finding on the greater imaging likelihood for cancer patients who died within 1 to 2 months of diagnosis conflicts with a US report,^11^ warranting further research. Similarly, our finding that cancer patients with more concurrent chronic diseases were significantly more likely to receive CT but less likely to receive MRI warrants further investigation, since the association of comorbidity status with high-tech/high-cost imaging use at EOL has never been explored.

The likelihood of receiving high-tech/high-cost imaging was generally less for subjects cared for by oncologists/hematologists than by other physician specialists. This finding may be related to differences in physicians’ EOL care experiences and beliefs about the effectiveness of imaging at EOL. Oncologists/hematologists have more frequent contact with dying patients^36^ and greater experience caring for terminally ill cancer patients than most other physician specialists, making them more likely to forgo life-sustaining treatments for terminal patients.^37^ By the same token, they may be less likely to use CT, MRI, and BS for their cancer patients at EOL.

We report a novel finding, that is, undergoing high-tech/high-cost imaging in Taiwanese cancer patients’ last month of life varied by their primary hospital's characteristics and EOL care practice patterns. Aggressive EOL care (i.e., ICU care, chemotherapy, and life-prolonging procedures) has been strongly linked to primary hospital teaching status^8^ and EOL care intensity at both the hospital^38^ and regional levels.^10,12^ We extend these findings by showing that cancer patients were more likely to be imaged at EOL if they received care at a teaching hospital and in a hospital (Figure 4II) or a region with higher EOL care intensity, outweighing the influence of traditional healthcare resource indicators (i.e., acute care beds).

Contrary to our expectation that using high-tech/high-cost imaging in Taiwanese cancer patients’ last month of life would be negatively associated with hospital case volume of terminal cancer patients, we found opposite trends with MRI and BS (Figure 4I). Volume–outcome relationships have been well-established at the hospital level for complicated cancer surgeries^39^ and mammography use for follow-up screening, MRI for low back pain, abdominal/thoracic CT,^34^ and EOL care quality, including avoiding ICU use at EOL.^24^ We did not find an inverse association between case volume of terminal cancer patients and high-tech/high-cost imaging use at EOL, suggesting that hospice/palliative care philosophy and practices have not diffused into imaging use at EOL in Taiwan.

The strengths of our study lie in its population-based approach and focus on a recent decade-long period. However, claims data lack the clinical information needed to evaluate indications for individual imaging procedures. Thus, our results likely overestimate cancer-specific rates of imaging use. We also did not evaluate the appropriateness of receiving high-tech/high-cost imaging at EOL by measuring associations between high-tech/high-cost imaging and subsequent management. Despite simultaneously investigating patient, physician, hospital, and regional characteristics and EOL care practice patterns in our adjusted analyses, we cannot exclude the possibility that our results are partly attributable to unmeasured patient characteristics, that is, preferences for imaging, functional dependence, and symptom distress; physician attitudes toward and incentives for using high-tech/high-cost imaging; and hospital microclimates or cultures. Finally, the effect sizes for some variables reached statistical significance due to the large study cohort but may have minimal clinical significance.

## CONCLUSION

A substantial proportion (36.11%) of Taiwanese cancer patients with metastatic disease received at least 1 of 4 high-tech/high-cost imaging modalities in their last month of life, and CT, MRI, and PET use increased significantly overtime. Further research is needed to characterize the appropriateness of high-tech/high-cost imaging use in EOL cancer care. Hospitals/physicians should be charged with greater accountability for reducing unnecessary high-tech/high-cost imaging at EOL, because they exert local control over decisions to use expensive technology.^40^ Nonmedical oncologists/hematologists affiliated with teaching hospitals and hospitals that tend to aggressively treat high volumes of at-risk terminally ill cancer patients should be targeted by clinical and financial interventions to help them carefully evaluate the effectiveness of using high-tech/high-cost imaging at EOL. By providing necessary, affordable, and sustainable imaging at EOL, the maximum benefits can be achieved not only for patient symptom relief and quality of life, but also for societal resources.
